# Differential activation of sporamin expression in response to abiotic mechanical wounding and biotic herbivore attack in the sweet potato

**DOI:** 10.1186/1471-2229-14-112

**Published:** 2014-04-28

**Authors:** SenthilKumar Rajendran, I-Winnie Lin, Mei-Ju Chen, Chien-Yu Chen, Kai-Wun Yeh

**Affiliations:** 1 Institute of Plant Biology, National Taiwan University, Taipei 106, Taiwan; 2 Genome and Systems Biology Degree Program, National Taiwan University and Academia Sinica, Taipei 106, Taiwan; 3 Department of Bio-Industrial Mechatronics Engineering, National Taiwan University, Taipei 106, Taiwan

**Keywords:** Sporamin, Different activation, Jasmonic acid, Salicylic acid, Transcription factors: NAC, WRKY, ROS, Transcriptome, Sweet potato

## Abstract

**Background:**

Plants respond differently to mechanical wounding and herbivore attack, using distinct pathways for defense. The versatile sweet potato sporamin possesses multiple biological functions in response to stress. However, the regulation of sporamin gene expression that is activated upon mechanical damage or herbivore attack has not been well studied.

**Results:**

Biochemical analysis revealed that different patterns of Reactive oxygen species (ROS) and antioxidant mechanism exist between mechanical wounding (MW) and herbivore attack (HA) in the sweet potato leaf. Using LC-ESI-MS (Liquid chromatography electrospray ionization mass spectrometry analysis), only the endogenous JA (jasmonic acid) level was found to increase dramatically after MW in a time-dependent manner, whereas both endogenous JA and SA (salicylic acid) increase in parallel after HA. Through yeast one-hybrid screening, two transcription factors IbNAC1 (no apical meristem (NAM), *Arabidopsis* transcription activation factor (ATAF), and cup-shaped cotyledon (CUC)) and IbWRKY1 were isolated, which interact with the *sporamin* promoter fragment of *SWRE* (*sporamin wounding-responsive element*) regulatory sequences. Exogenous application of MeJA (methyl jasmonate), SA and DIECA (diethyldithiocarbamic acid, JAs biosynthesis inhibitor) on sweet potato leaves was employed, and the results revealed that IbNAC1 mediated the expression of *sporamin* through a JA-dependent signaling pathway upon MW, whereas both IbNAC1 and IbWRKY1 coordinately regulated *sporamin* expression through JA- and SA-dependent pathways upon HA. Transcriptome analysis identified *MYC*2/4 and *JAZ*2/*TIFY*10A (jasmonate ZIM/tify-domain), the repressor and activator of JA and SA signaling among others, as the genes that play an intermediate role in the JA and SA pathways, and these results were further validated by qRT-PCR (quantitative real-time polymerase chain reaction).

**Conclusion:**

This work has improved our understanding of the differential regulatory mechanism of *sporamin* expression. Our study illustrates that sweet potato *sporamin* expression is differentially induced upon abiotic MW and biotic HA that involves IbNAC1 and IbWRKY1 and is dependent on the JA and SA signaling pathways. Thus, we established a model to address the plant-wounding response upon physical and biotic damage.

## Background

Plants sense and respond to external stimuli using a repertoire of mechanisms that regulate gene expression for survival in hostile environments. To defend against external stimuli, plants have evolved inducible defense mechanisms against microbial pathogens and herbivores that involve the regulation of gene expression for the synthesis of specific proteins and secondary metabolites [1]. Signals that mediate systemic plant responses are classified as either slow or fast moving, travelling within minutes to several hours, and are typically mediated by different hormones/electrical signals [2]. These fascinating phenomena imply the existence of cell-cell communication that transmits the defense response over a long distance [3]. Wounding responses in plants regulate multiple signaling pathways [4,5]. It is well established in tomatoes and *Arabidopsis* that JA is a systemic, long-distance, mobile- signaling molecule that functions by transmitting information about wounding to distant, non-wounded tissues where a defense response is invoked [3,6]. Moreover, jasmonates, which are synthesized from α-linolenic acid (LA, 18:3) via the octadecanoid pathway, induce the expression of a wide range of defense genes against MW, HA and necrotrophic pathogens [2,7]. Mutant and traditional grafting experiments support that the JA-signaling pathway creates long-distance mobile signals to activate defense gene expression [8]. In addition, there is sufficient evidence that the kinetics of the JA and SA signaling pathways aid in choosing the defensive strategy following stress [9,10]. Cross-talk between phytohormones other than JA and SA, ethylene and ABA (abscisic acid) has not been extensively studied with respect to wounding signal perception (for reviews, see [11,12]). It has been well documented that signal transduction pathways often overlap and that the induced responses to various stimuli are different [13]. For instance, insects chewing cause severe damage to leaf tissue and release volatile organic compound (VOC), which induce direct defense by activating wound signaling pathway [5]. Some of the oxylipin compounds (JA) have the ability to be a master-switch against herbivores and turn on herbivore-related defense genes [14]. Moreover, saliva-derived compounds are brought to the wound site during HA. These saliva-derived compounds (FAC -fatty acid amino acid conjugate) were shown to induce an outburst of both JA and herbivore-induced volatile organic compound (HI-VOC) [15,16]. How the herbivore feeds on the plant tissue, either by sucking or chewing, will determine whether the signaling pathway is JA solely, or JA and SA, induced [16,17]. The coevolution between plants and herbivores has generated a costly defensive trait, which is greatly dependent on the concentration and timing of the hormone (JA, SA, or ethylene) [17,18]. Extensive studies defining the global expression profiles upon the activation of wounding signaling pathways have been conducted using microarrays [19] and proteomic [20]. The role of such signaling hormones, integrated in the decision-making of stress response, are played by a class of Reactive oxygen species (ROS) [21]. An example of ROS as retrograde signaling from chloroplast to nucleus having sub-cellular and systemic functionality has been documented [22,23]. However, these cells are equipped with an excellent antioxidant (enzymatic or non-enzymatic) defense mechanism to detoxify the harmful effects of ROS [24]. Sweet potato (*Ipomoea batatas*) with its hexaploid complex genome (2n = 6× = 90) accounts for the fifth-largest tuber crop worldwide. Unlike others, the cultivar Tainong 57 has great potential to defend itself against herbivore and/or wounding stress. The versatility of sweet potato sporamin makes it a very attractive tuberous storage protein because of its strong trypsin inhibitory activity and multiple biological functions against various stresses [25].

In this article, we analyzed the first line of ROS scavenging mechanisms by antioxidant enzymatic activity in leaves after MW and HA. We dissect the role phytohormones play in shaping the interaction between MW and HA in the regulation of *sporamin* expression. We report for the first time that the sweet potato leaf, damaged by MW and HA, utilizes different phytohormone signals to orchestrate the interactive role in the regulation of the *sporamin* gene. Realizing the potential importance of these signals, we studied the regulatory mechanisms that attenuate two wound-induced transcription factors (IbNAC1 and IbWRKY1) by either JA or SA under the stress of MW and HA. Furthermore, we performed a transcriptome analysis of wounding stress and surveyed global gene expression to identify the relevant genes of the phytohormone network. Together, our findings illustrate the precise role of the phytohormone and signaling pathways that play critical roles in regulating sweet potato sporamin expression upon abiotic mechanical damage and biotic HA.

## Results

### *In vitro* antioxidant scavenging enzymatic activities after MW or HA

**Figure 1:**
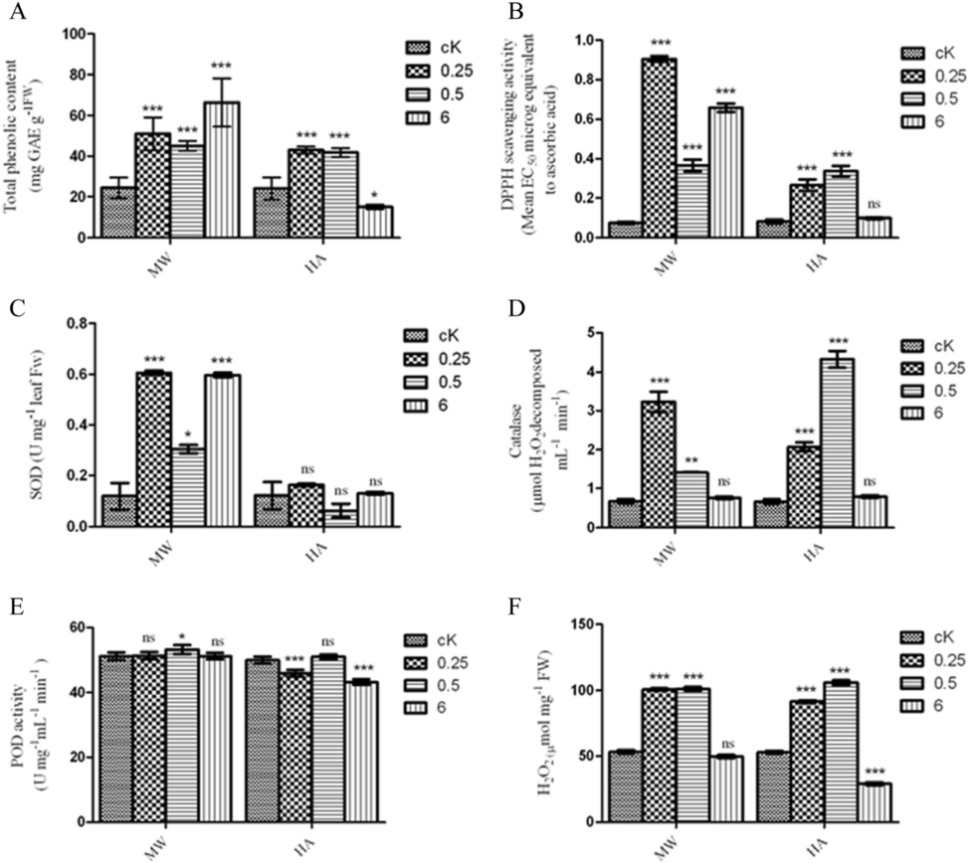
***In vitro *antioxidant studies of sweet potato leaves damaged by MW and HA.** The third leaves of sweet potato plants were sampled at 0.25, 0.5 and 6 h after treatment with MW or HA. **(A)** The total phenolic content was measured at 725 nm and is expressed in gallic acid (GAE) equivalents; **(B)** The DPPH activity was measured at 517 nm using ascorbic acid as a standard, and the EC_50_ was calculated; **(C)** The SOD activity was measured at 560 nm and defined as the amount of enzyme that resulted in 50% NBT inhibition; **(D-F)** The catalase activity **(D)**, POD activity **(E)** and H_2_O_2_ level **(F)** were measured using the TMB (3,3′,5,5′-tetramethylbenzidine) method. Data are means (±SE) of three independent experiments with three replicates. (* represents P < 0.05; ** represents P < 0.01; *** represents P < 0.001; ‘ns’ represents P > 0.05). DPPH: 2, 2-diphenyl-1-picrylhydrazyl; POD: peroxidase; SOD: superoxide dismutase.

Initial attempts were made to learn how the different ROS levels at the site of damage leads to increasing anti-oxidative enzymes and response to stress. Damage caused by free radical-induced oxidative stress is repaired by antioxidant enzymes, which are important as they protect cells. We performed two different stresses, MW and HA, to estimate the total level of antioxidant enzymes. The time points were designed to identify the early (0.25 h, 0.5 h) and late (6 h) responses of wounding stress in the sweet potato leaf. Phenolics can directly scavenge molecular ROS to eliminate free radicals, linearly correlated with antioxidant capacity. Total phenolic content and 1, 1-diphenyl-2-picrylhydrazyl (DPPH) assay was measured for control leaf (cK), leaf damaged by MW and leaf damaged by HA. The total phenol content from MW leaves increases at 0.25 h (51 ± 8.02), 0.5 h (45.1 ± 2.3), and 6 h (66.4 ± 11.8) compared to that of cK leaves (24.4 ± 5.08) (Figure 1A). The increase in phenolic content in response to insect feeding (HA) (cK, 24.1 ± 4.42; 0.25 h, 43 ± 1.78; 0.5 h, 41.7 ± 2.2; 6 h, 15. ±1.05) was likely not significant except for values 6 h time point (Figure 1A). In addition, the sweet potato leaves showed a significant increase in DPPH antioxidant level in response to MW and HA. Using the EC_50_ value of ascorbic acid (R^2^ = 0.9715), the DPPH antioxidant level was 0.90 ± 0.025 μg/mL, 0.36 ± 0.052 μg/mL and 0.65 ± 0.036 μg/mL at 0.25, 0.5 and 6 h, respectively, in response to MW (Figure 1B). Similarly, the DPPH antioxidant level was 0.26 ± 0.05 μg/mL, 0.33 ± 0.047 μg/mL and 0.09 ± 0.07 μg/mL at 0.25, 0.5 and 6 h, respectively, in response to HA. Collectively, a direct correlation between total phenolic content and DPPH free radical scavenging activity was shown in response to MW and HA at all the time points tested. However, a significant reduction of the total phenolic content and DPPH antioxidant level was observed upon HA as compared to MW.

Antioxidant-catalyzing enzymes (Superoxide dismutase (SOD), Catalase (CAT), Peroxidase (POD), and hydrogen peroxidase (H_2_O_2_)): The superoxide dismutase (SOD) activity increased at all of the time points tested in response to MW and HA, compared to the control (cK) (Figure 1C). The mean enzymatic activity of sweet potato SOD significantly increased at 0.25 h (0.60 U/mL) and 6 h (0.59 U/mL) after MW (Figure 1C). However, the enzymatic level at 0.5 h (0.30 U/mL) after MW was lower than that at 0.25 and 6 h. Similar enzymatic activity levels were observed at 0.25 h (0.16 U/mL) and 6 h (0.12 U/mL) after HA, but in comparison with MW, HA resulted in lower SOD activity (Figure 1C). Catalase assay: Catalase (CAT) is an enzyme that reduces oxidative stress by removing H_2_O_2_. Our results showed that in response to MW, the activity of catalase decreased significantly over time; 0.5 h (1.42 ± 0.01EA U/mL) and 6 h (0.75 ± 0.06 EA U/mL) was lower than that at 0.25 h (3.2 ± 0.45 EA U/mL) (Figure 1D). In contrast, upon HA, the catalase enzyme activity was significantly reduced at 0.25 h (2 ± 0.19EA U/mL) but increased at 0.5 h (4.3 ± 0.36EA U/mL) (Figure 1D). However, at 6 h (0.79 ± 0.06EA U/mL), some activity remained in both the MW and HA leaves (Figure 1D). Overall, this data indicates a significant increase in catalase activity in MW and HA leaves compared with control leaves (cK) (0.7773 ± EA U/mL). Peroxidase activity assay (POD): Hydrogen peroxide production often results in an increase in peroxidase activity in response to stress. From our data, the peroxidase activity did not show any significant differences between the control leaves and the MW and HA leaves at the different time points (Figure 1E). However, a notable increase in the basal level activity was observed in the MW leaves. In contrast, the HA leaves showed a slight decrease in peroxidase activity at all of the time points tested (Figure 1E). Hydrogen peroxidase activity assay (H_2_O_2_): Next, we used the TMB (3,3′,5,5′-tetramethylbenzidine) assay to determine the hydrogen peroxide scavenging activity, which revealed notable changes in this activity between control leaves and MW or HA leaves. The H_2_O_2_ scavenging activity at 0.25 and 0.5 h was 101.7 ± 1.72 μM and 101.1 ± 2.37 μM upon MW, respectively, compared to the control (53.3 ± 2.41 μM) (1 F). However, the H_2_O_2_ scavenging activity gradually decreased in both the MW (49.6 ± 2.06 μM) and HA (28.8 ± 1.95 μM) leaves at 6 h (1 F). Collectively, our results of total phenolic content (Figure 1A), DPPH antioxidant level (Figure 1B), POD (Figure 1E) and H_2_O_2_ assay (Figure 1F) showed similar or identical total antioxidant level and activity upon MW and HA stresses. In contrast, SOD (Figure 1C) and CAT (Figure 1D) showed a significantly different pattern of total antioxidant level and activity upon MW and HA stresses.

### *In vivo* fluorescence determination of the singlet oxygen species (^1^O_2_) levels in response to wounding stress

**Figure 2:**
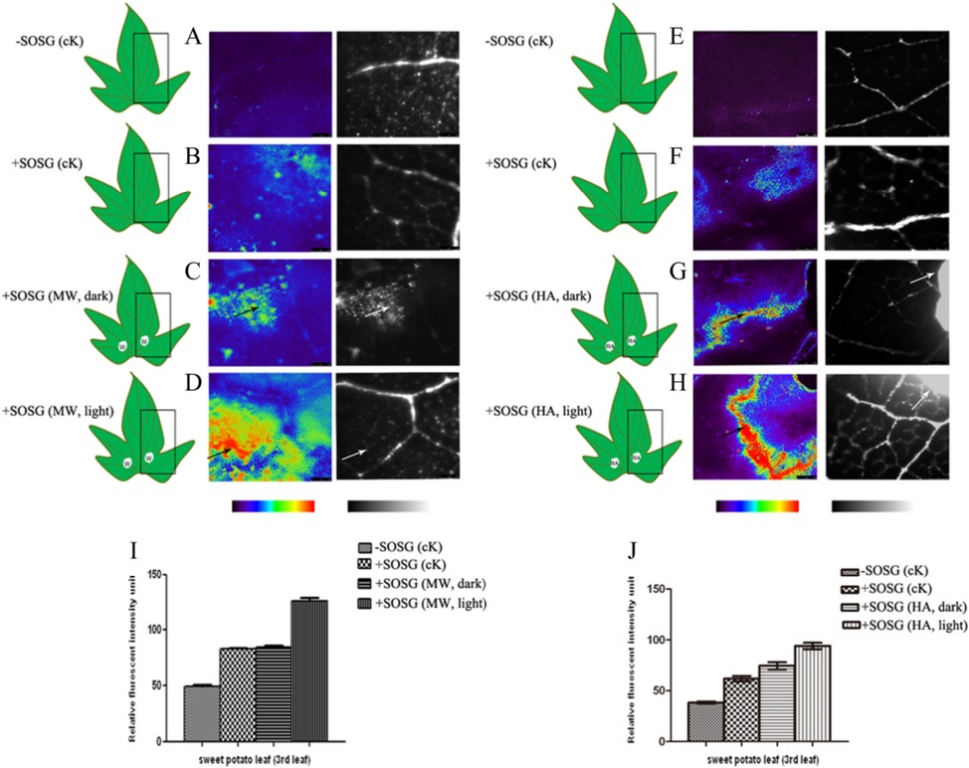
**Wound-induced production of **^**1**^**O**_**2 **_**in sweet potato leaves.** Confocal laser-scanning microscopic (CLSM) images of sweet potato leaves. False color image of sweet potato leaf infiltrate with SOSG and exposed to light (150 μmol m^-2^ s^-1^ PPFD) for 15 min. **(A, E)** Control leaf (cK) without wounding and SOSG treatment that was kept in the dark for 12 h. **(B, F)** Control leaf infiltrated with SOSG and kept in dark for 15 min. **(C, G)** Wounded leaf (MW, HA) infiltrated with SOSG and exposed to dark for 15 min. **(D, H)** Wounded leaf (MW, HA) infiltrated with SOSG and exposed to light for 15mins. **(I, J)** Relative quantification of image inflorescence by Image J. The circle denotes the wounded area. The black arrow indicates the production of ^1^O_2_. The SOSG fluorescence was measured using an excitation wavelength of 525 nm. SOSG: singlet oxygen sensor green; W: wounded; cK: non-wounded.

To assay the singlet oxygen (^1^O_2_) level at an early time point is essential because it is the primary signal to induce other ROS species. Here, we monitored wounded leaves (both MW and HA) under the short illumination duration (0.25 h) and light intensity. The formation of ^1^O_2_ was measured by SOSG dye (singlet oxygen sensor green). Figure 2A-H shows our confocal laser-scanning microscope (CSLM) experiment of leaves from unwounded (cK) and MW or HA leaves (0.25 h) that were infiltrated with SOSG dye in the dark or light. Substantial SOSG fluorescence was only detected in MW or HA leaves treated with SOSG in the dark and in the light compared to the cK leaf (Figure 2C, D and G, H). The significant generation of ^1^O_2_ in sweet potato leaves damaged by MW or HA induces hydrogen peroxide and superoxide in the presence of light, suggesting the cross-talk role of ^1^O_2_ production during ROS metabolism (Figure 2).

### Alteration of endogenous JA and SA levels in sweet potato leaves in response to MW or HA

**Figure 3:**
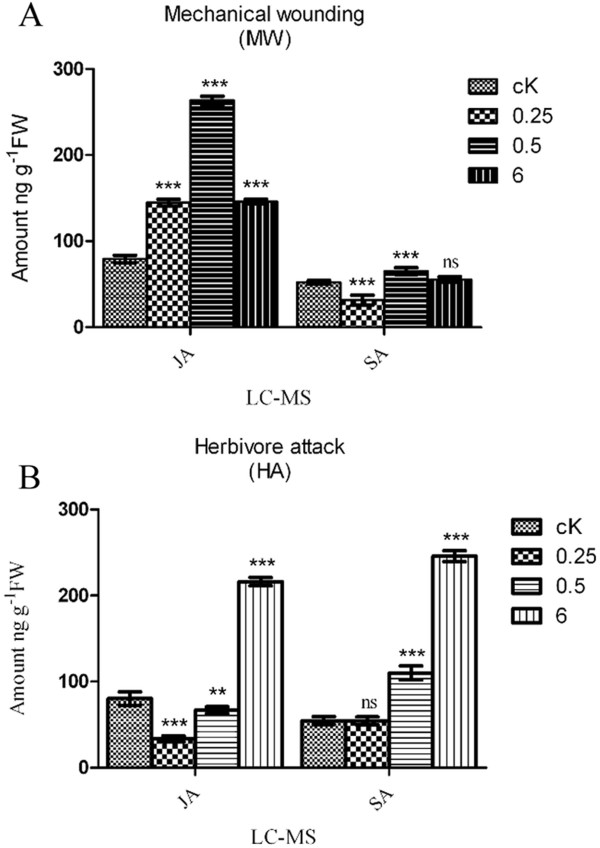
**LC-ESI-MS quantification of the endogenous phytohormones JA and SA in sweet potato leaves.** The healthy third leaf of a sweet potato plant without treatment was used as a control (cK). The JA and SA levels were quantified at 0.25, 0.5 and 6 h after the third leaf was damaged by: **(A)** MW; or **(B)** HA (second instar larvae of *Spodoptera littoralis*). The level of JA and SA was expressed as ng JA/SA per g FW. (* represents P < 0.05; ** represents P < 0.01; *** represents P < 0.001; ‘ns’ non-significant represents P > 0.05). Values are the means (±SE) of three replicates (n = 6).

From the changes of ROS levels, we suspected that leaves damaged by MW or HA (*Spodoptera littoralis*) eventually increase endogenous hormones for defense signaling. To address this point, a time-course analysis (0.25, 0.5 and 6 h) was carried out to quantify the levels of endogenous JA and SA by LC-ESI-MS in the third leaf of sweet potato plants damaged by MW or HA. In a healthy sweet potato leaf, the endogenous levels of JA and SA were 79.4 ± 4.3 ng/g fresh weight (FW) and 52.3 ± 1.9 ng/g FW, respectively (Figure 3A). In the leaf damaged by MW, the level of endogenous JA began to increase at 0.25 h and remained significantly higher than its initial level at 0.5 h (Figure 3A). This increase in the endogenous JA level was 2-fold higher than the control (cK). In contrast, the level of endogenous SA began to increase slightly at 0.5 h and was significantly reduced at 0.25 h (Figure 3A). At 6 h after MW, both endogenous JA and SA were detected at their basal levels (Figure 3A). In contrast, upon HA, the level of endogenous JA positively correlated with MW at 0.25 and 0.5 h and at 6 h (2-fold) (Figure 3B). Similarly, we noticed a significant and stable increase in the SA level beginning at 0.5 h (2-fold) and continuing to 6 h (4-fold) after HA (Figure 3B). Thus, our results indicate that the levels of JA and SA are inversely correlated during mechanical damage and HA in sweet potato leaves. The reduction of SA and JA (Figure 3A and B) could be explained by the variation between the samples tested.

### NAC and WRKY were the DNA-binding proteins that recognize *SWRE* element of sporamin promoter

**Figure 4:**
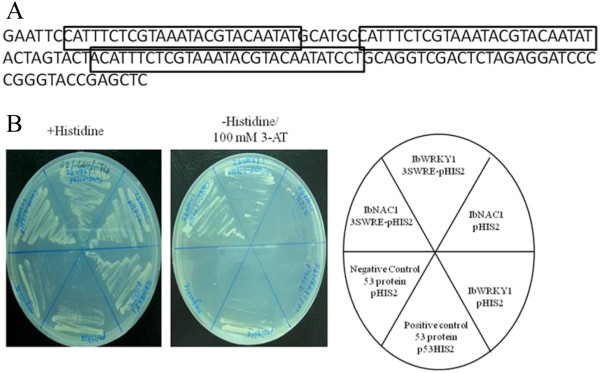
**Isolation of Ib*NAC*1 and Ib*WRKY*1 proteins interacting with SWRE DNA by yeast one-hybrid screening.** Two cDNA clones (*IbNAC1* and *IbWRKY1*) were isolated by Y1H screening, and re-transformed into the yeast strain Y187 in the presence of plasmid containing three tandem repeats of the *SWRE* sequence (*3*x*SWRE*-p*HIS*2) **(A)** or without *3*x*SWRE*3 (p*HIS*2). Growth of yeast transformants is shown on both + His **(A)** and -His/100 mM 3-AT medium **(B)**. Positive control and negative control are employed to standardize selection condition by using yeast strain Y187 co-transformed with p*GAD*-*Rec*2-53/p53*HIS*2 or p*GAD*-*Rec*2-53/p*HIS*2 (Clontech).

A yeast one-hybrid screening system was performed to isolate a DNA-binding protein that binds to three tandem repeats of the *SWRE* fragment (*3*x*SWRE* (38-bp), -1103- ACATTTCTCGTAAATACGTACAATATCCTTGTCTTTCC -1061). A cDNA library generated from wounded sweet potato leaves was expressed as a translational fusion with a *GAL4* activation domain (AD/cDNA library) in a yeast reporter strain which carries an integrated *His3* allele, with the three tandem copies of the *SWRE* sequence (*3*x*SWRE*) employed as a probe (Figure 4A). At the first screening using the library, which contains 6 × 10^5^ cfu, 94 colonies were selected from the selective medium and were re-streaked on a more stringent selective medium. Finally, two cDNAs, named Ib*NAC*1 (Accession: GQ280387) and Ib*WRKY*1 (Accession: GQ280386) respectively, emerged within the *SWRE* fragment sequence. In order to confirm their binding ability with *SWRE* fragments, these two clones were transformed into a yeast cell containing the p*HIS2*-*3*x*SWRE* reporter vector or p*HIS2* as a negative control again. As shown in Figure 4B, co-transformation of *pGADT7-Rec2*-Ib*NAC*1 with p*HIS2*-*3*x*SWRE* or p*HIS2* can both grow in the SD/-Leu/-Trp medium, as well as p*GADT7*-*Rec2*-Ib*WRKY*1 with p*HIS2*-*SWRE* or p*HIS2*. However in the SD/-His/-Leu/-Trp plus 100 mM 3-AT selective medium, only co-transformation of p*GADT7*-*Rec2*-Ib*NAC*1 or p*GADT7*-*Rec2*-Ib*WRKY*1 with p*HIS2*-*3*x*SWRE* can grow but not p*HIS2* reporter vectors only (Figure 4).

### The regulation of sporamin is facilitated by transcription factors in response to wounding stress and hormonal treatment

**Figure 5:**
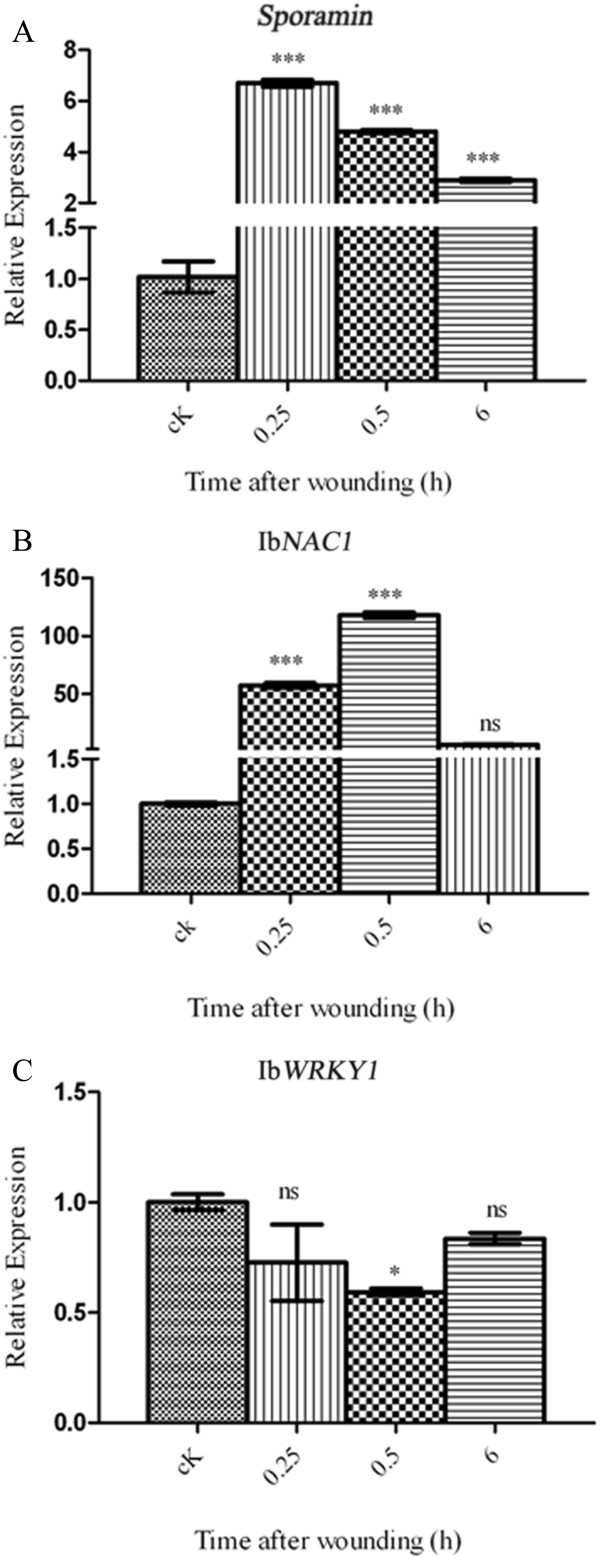
**QRT-PCR analysis of the sporamin, Ib*NAC*1 and Ib*WRKY*1 transcript after different wounding treatments.** Relative expression of *sporamin*, Ib*NAC*1 and Ib*WRKY*1 expression were measured by qRT-PCR in untreated (cK) and mechanically wounded (MW) sweet potato leaves at the indicated time points. *Ubiquitin* (*UBQ*) gene was used as an internal control. (* represents P < 0.05; ** represents P < 0.01; *** represents P < 0.001; ‘ns’ not significant represents P > 0.05). The results are representative of three replicates.

To address whether the systemic expression of *sporamin*, Ib*NAC*1 and Ib*WRKY*1 gene were activated by endogenous hormones JA and SA in response to wounding, we executed an experiment in which similar time points were analyzed. QRT-PCR was performed on sweet potato leaves treated subjected to MW and treated with the exogenous application of MeJA, SA or DIECA. We reproducibly demonstrated that the expression of *sporamin* transcript is induced in leaves damaged by MW over the time course (Figure 5A). Similarly, the expression of Ib*NAC*1 was strongly induced at 0.25 and 0.5 h and at 6 h (Figure 5B) after leaves damaged by MW.

On the other hand, the accumulation of the Ib*WRKY*1 transcript did not show any induction at all of the time points tested (Figure 5C). Thus, *sporamin* and Ib*NAC*1 were induced upon MW stress, suggesting the association of Ib*NAC*1 as a positive regulator and Ib*WRKY*1 as a negative regulator of *sporamin* gene expression upon MW stress.

**Figure 6:**
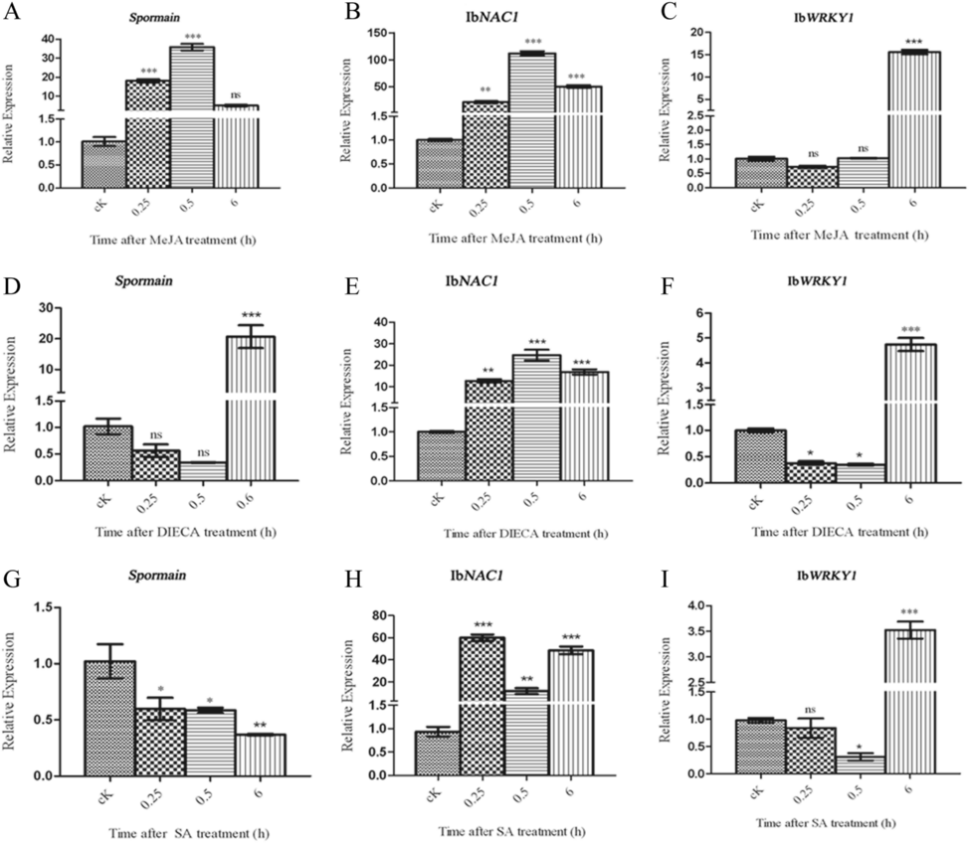
**Expression profiles for sporamin, Ib*NAC*1 and Ib*WRKY*1 in sweet potato leaves.** The third leaves of sweet potato plants were sampled at 0.25, 0.5 and 6 h after treatment with MeJA (0.5 μM) **(A-C)**, DIECA (50 mM) **(D-F)**, or SA (2 mM) **(G-I)** independently. Total RNA was extracted at the indicated time points from untreated (cK) and treated leaf samples. **(A-I)** Relative expression of *sporamin*, Ib*NAC*1 and Ib*WRKY*1 transcripts were measured by qRT-PCR. Asterisks indicate statistically significant differences (Dunnett’s multiple comparison test, *represents P < 0.05; ** represents P < 0.01; *** represents P < 0.001; ‘ns’ not significant represents P > 0.05). The results are representative of three replicates.

Next, we examined the expression level of the *sporamin*, Ib*NAC*1 and Ib*WRKY*1 gene after the application of MeJA. Using qRT-PCR, we confirmed that the *sporamin* and Ib*NAC*1 transcripts induced at 0.25 and 0.5 h after the exogenous application of MeJA, and their level noticeably declined from 0.5 to 6 h (Figure 6A and B). Furthermore, Ib*WRKY*1 was not detected at the early time points (0.25 and 0.5 h) and showed a remarkably higher expression at 6 h after MeJA treatment (Figure 6C). In plants, DIECA is a potent inhibitor of JA- biosynthesis in the octadecanoid pathway [26]. As expected, exogenous application of DIECA was only effective against the expression of *sporamin* transcript at the early time points 0.25 and 0.5 tested, and had no effect at 6 h after treatment (Figure 6D). However, exogenous application of DIECA does not alter the Ib*NAC*1 gene expression (Figure 6E). In contrast, Ib*WRKY*1 transcript had no expression at early time points, similar to the without-DIECA treatment (Figure 5C), but induced to express only at 6 h after DIECA treatment (Figure 6F). Together, this can be easily explained by the fact that blocking of JA biosynthesis will automatically lower the signal transduction in the JA-signaling pathway; antagonistically, SA levels should be induced and SA-responsive genes should be up-regulated.

Furthermore, upon treatment with SA, our qRT-PCR results shows that the low level or basal expression of *sporamin* was significant at all the time points tested (Figure 6G). On the other hand, strong activation of Ib*NAC*1 gene expression was stable post-exogenous application of SA treatment at all the time points (Figure 6H). Thus, we hypothesize IbNAC1 is a positive convergence regulator, activated upstream of *sporamin* that is influenced by both JA- and SA-mediated signaling in a time-dependent manner. Moreover, the accumulation of Ib*WRKY*1 did not show any expression at early time points (0.25 and 0.5), but induced to express at 6 h after SA treatment (Figure 6I). Therefore, Ib*WRKY*1 has a partial role in both JA and SA signaling at later time points in the wound- signaling pathway.

### Effects of HA on the expression of *sporamin* and the transcription factors Ib*NAC*1 and Ib*WRKY*1 in sweet potato leaves

**Figure 7:**
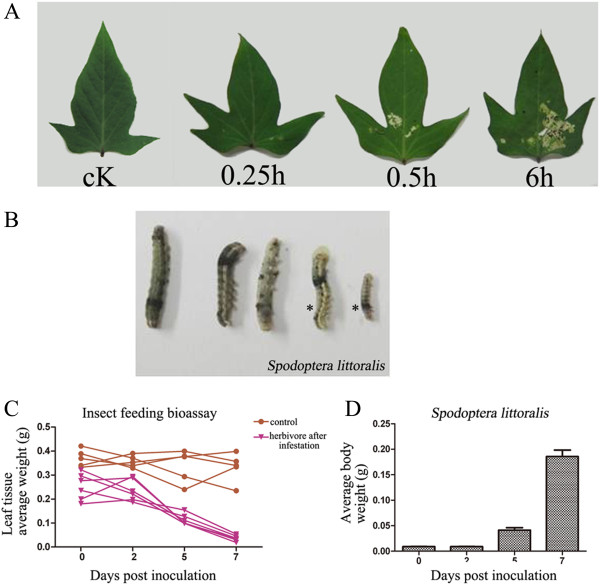
**Insect bioassay. (A)** The mass of leaves consumed by herbivores (*Spodoptera littoralis*) at the indicated time points; **(B-C)** The mean mass (±SE) of second instar larvae of *Spodoptera littoralis* reared on sweet potato leaves (n = 10) and the plant growth biomass were recorded after 7 days **(B)** and from 2–7 days **(C)** of feeding **(D)**. The results are representative of three replicates. (* represents growth impairment of larvae).

To demonstrate that leaves damaged by HA have different responses to those with MW, second instar larvae (starved for 2 days) were placed on the underside of every third leaf of sweet potato plants and the leaves were observed for biotic damage. Herbivore (*Spodoptera littoralis*)-eaten leaves were collected at three different time points (0.25, 0.5 and 6 h), and the mass of the leaves consumed by the larvae was moderately reduced at 6 h after feeding began (Figure 7A). We further observed the behavior and survival of the herbivores, and our results showed that the body color of the larvae changed from brown to brownish-green and the larvae moved from the upper side to the lower side of the leaves whenever perturbed. Furthermore, the growth of the larvae was measured every 2 days for a period of 7 days. A few of the larvae died at various stages, leaving 6–8 individuals that survived to adults (Figure 7B). Their weight moderately increased, beginning at day 4 and continuing to increase until day 7 (Figure 7C). The larvae produced wet fecula from the dorsal anus from day 1 to day 3 (data not shown).

**Figure 8:**
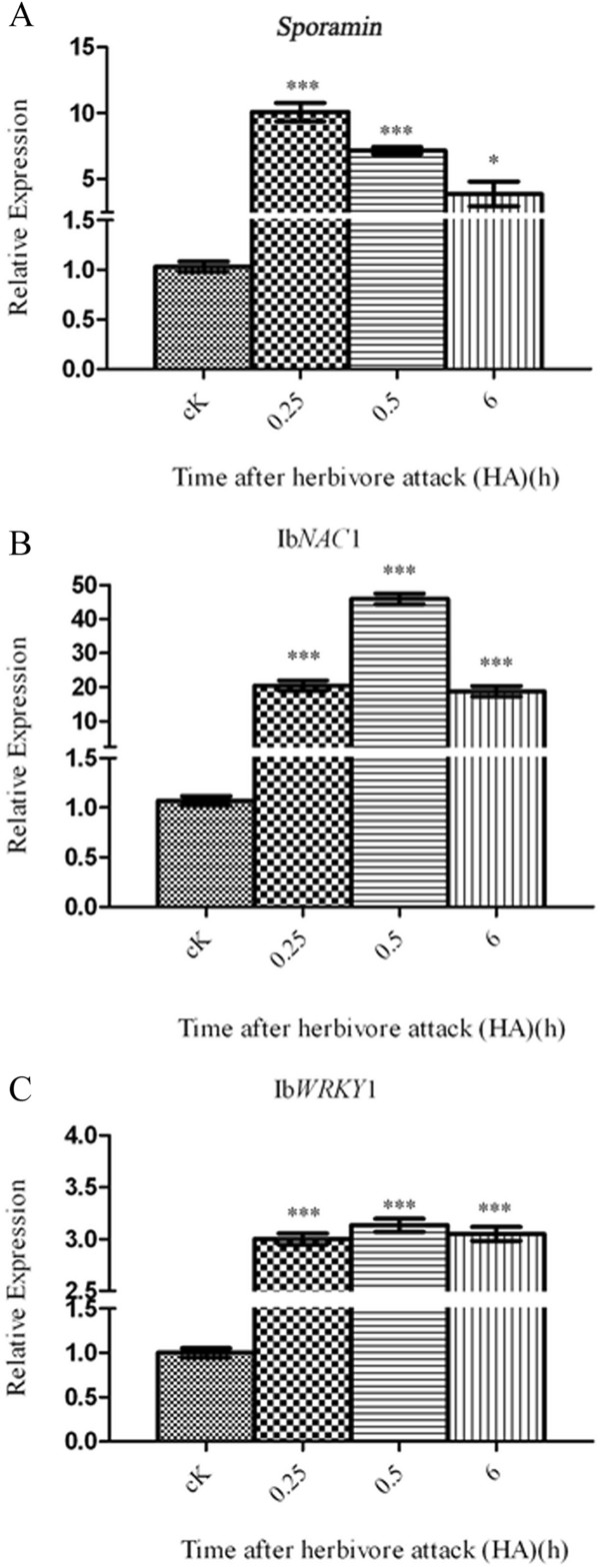
**HA and gene expression profiles for *sporamin*, Ib*NAC*1 and Ib*WRKY*1 in sweet potato leaves. (A-C)** Relative expression of *sporamin*, Ib*NAC*1 and Ib*WRKY*1 transcripts were measured by qRT-PCR analysis in untreated leaves (cK) and leaves damaged by HA. The results are representative of three replicates. Asterisks indicate statistically significant differences. (Dunnett’s multiple comparison test,* represents P < 0.05; ** represents P < 0.01; *** represents P < 0.001; ‘ns’ not significant represents P > 0.05). The results are representative of three replicates.

Next, we examined the expression of *sporamin*, Ib*NAC*1 and Ib*WRKY*1 in response to HA by qRT-PCR. We observed a similar pattern of *sporamin* and Ib*NAC*1 gene expression between MW (Figure 5A and B) and HA (Figure 8A and B). However, we did find a significant accumulation of Ib*WRKY*1 transcripts in leaves damaged by HA (Figure 8C). In the HA leaf, the accumulation of Ib*WRKY*1 began at 0.25 h, peaked at 0.5 h and was maintained until 6 h (Figure 8C). This differs from leaves with MW (Figure 5C), in which the transcript levels of Ib*WRKY*1 transcription factor was inversely expressed. Collectively, we concluded that endogenous JA plays a major role in the activation of *sporamin* gene expression upon two different stresses (MW or HA) mediated by Ib*NAC*1. However, the differentiated responses of Ib*WRKY* through *S. littoralis*-specific oral secretion created a cross-talk interaction of JA and SA signaling pathways.

### Solexa sequencing: global expression profiling of the sweet potato response to MW

#### Digital gene expression library sequencing

**Figure 9:**
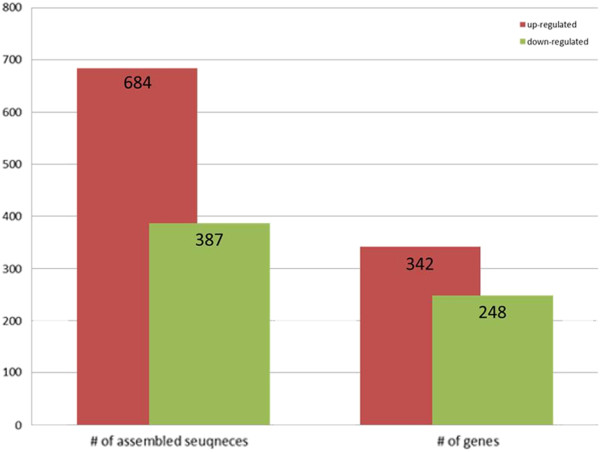
**Differential gene expression profiling.** The genes that were up- and down-regulated in response to wounding.

**Figure 10:**
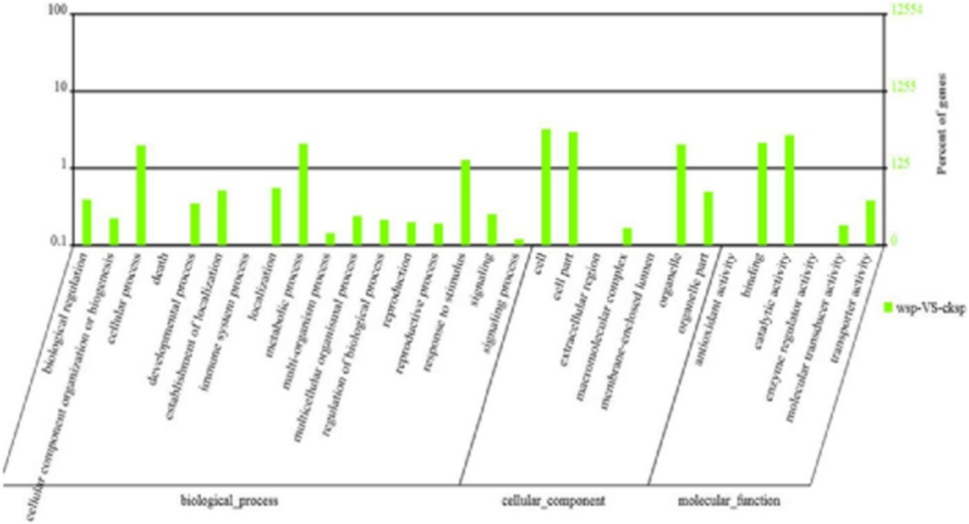
GO classifications of DEGs in sweet potato leaves damaged by MW and categories pertaining to biological process, cellular component and molecular function.

In this study, we conducted Solexa sequencing from sweet potato leaf (third leaf) with MW (15mins) and control without wounding (cK). A total 41,237,622 million raw reads were produced by using the llumina Solexa Genome Analyzer (GA) II sequencing platform, with total nucleotides 2, 784,289,408 (10 MB). The mean read length, contig length and GC % were 75.00, 519 bp and 44.74% for control and 76.15, 493 bp and 44.70 for wounding library. Distribution of total clean reads and its coverage are shown (Additional file 1: Figure S1). After quality trimming, the removal of primers, adaptor, poly (A) sequences, and low quality reads, we obtained a total of 20,777, 585 and 16,098,635 reads from MW and cK libraries, respectively (Additional file 2: Table S1). A total of 36,876,220 reads were obtained from the wounding and control libraries (Additional file 3: Table S2). A total of 41,806 differentially expressed transcripts were identified from control and wounding libraries. We have analyzed the most differentially regulated transcripts with a log2ratio >1 or < -1 as a threshold by means of significant value (P < 0.001) as well as false discovery rates (FDR < 0.01), representing 1,070 differentially expressed (684 up- and 386 down-regulated) transcripts (Figure 9) (Additional file 4). The major GO (Gene Ontology) terms corresponding to the differentially expressed transcripts are shown in Figure 10. A large number of transcripts were associated with ‘response to stimulus’, ‘signaling’, ‘hormone response’, ‘metabolic’ and ‘cellular process’. Among the differentially expressed genes (DEGs), we observed that a large number of transcripts were associated with responses to stimulus such as *allene oxide synthase* (*AOS*), *alternative oxidase* (*AOX*), *sensitive to proton rhizotoxicity* (*STOP1*), *SEN1*, *salt tolerance zinc finger* (*STZ*), *non*-*race specific disease resistance* (*NDR1*) and *nucleosome assemble protein*- *related protein NRP1*, including *sporamin* (*SPOR*), *ipomoein* (*IPO*), etc. Furthermore, we noticed that the several transcription factors, such as *Arabidopsis transcription activator factor* (*ATAF2*), *WRKY*, *Arabidopsis response regulator* (*ARR*), *ethylene responsive factor* (*ERF*), *MYC* etc., overlap in the subcategory of ‘response to stimulus’.

### Enriched pathway analysis of DEGs

**Figure 11:**
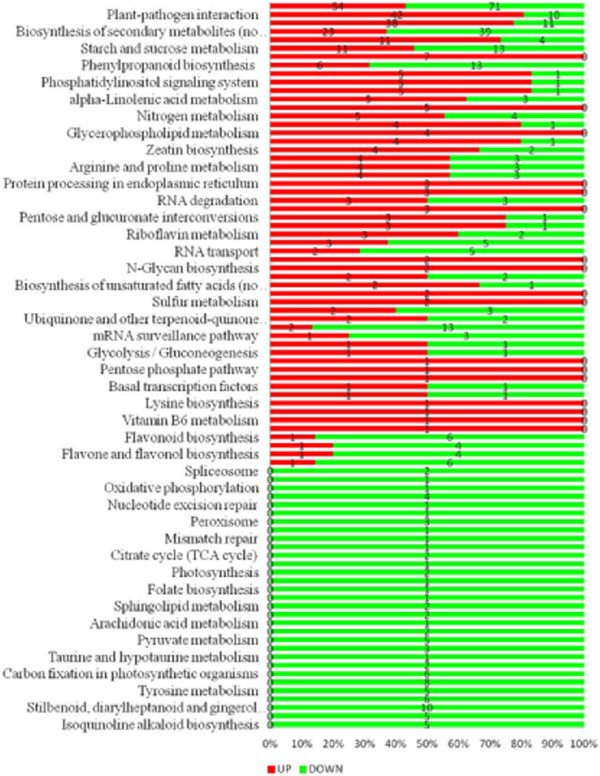
KEGG pathways of DEGs responding to wounding stress.

In this study, the DEGs were mapped to a total of 89 Kyoto Encyclopedia of Genes and Genomes (KEGG) pathways with Q value of <0.05 (Figure 11). The 15 most enriched pathways were involved in ‘phenylalanine metabolism’, ‘starch and sucrose metabolism’, ‘nitrogen metabolism’, ‘plant-hormone signal transduction’, ‘amino sugar nucleotide sugar metabolism’, ‘plant-pathogen interaction’, ‘phenylpropanoid biosynthesis’, ‘alanine, aspartate and glutamate metabolism’ and ‘biosynthesis of secondary metabolites’. Altogether, these results shed light on the wounding response in the sweet potato leaf, which is overwhelmed by dynamic metabolism as well as actively adapting to the hostile environment (Figure 11).

### Gene enrichment analysis

Next, GO term enrichment was analyzed using BiNGO software ( http://bingoware.sourceforge.net/). We next screened and compared the DEGs against the *Arabidopsis* genome database. Of the 1,070 differentially expressed transcripts, 809 (497 up- and 312 down-regulated) transcripts were matched to the *Arabidopsis* genome with an E-value 10^-10^. Of these 809 differentially expressed transcripts, 590 genes were found to have putative biological functions (Additional file 3: Table S2). These 590 genes were assigned to various functional role categories based on their significant matches to proteins that were already assigned a functional role. Their expression patterns were separated into six major clusters based on their gene enrichment values. Transcripts from each cluster are presented in the Additional file 5: Figures S2 and Additional file 6: Figure S3.

### Quantitative real-time PCR (qRT-PCR) confirming accuracy of Solexa sequencing

**Figure 12:**
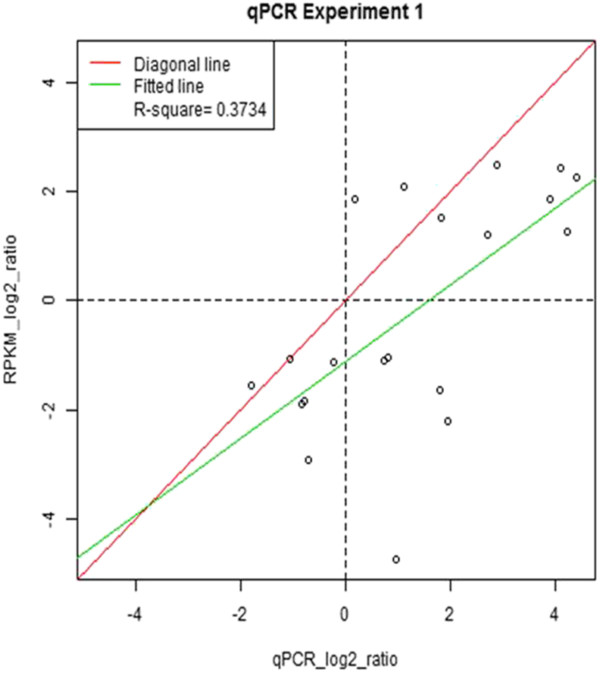
**Validation of transcriptome data by qRT-PCR.** Genes were selected based on gene enrichment analysis of the wounding stress (0.25 h) condition. Quantitative real-time PCR analysis was performed for 15 genes, and the values (least squares means) were fitted to a linear regression model. Both the x- and y-axes are shown in a log_2_ scale. Real-time PCR data was obtained from three independent experiments. *Actin* (*Act2*) was used as an internal control.

The expression of all 15 DEGs was consistent with the predictions based on the Illumina sequencing results. Among them, nine genes (*alpha-glucan phosphorylase 2; UDP-D-glucuronate 4 epimerase 1; Ethylene-responsive transcription factor 3; Tetraspanin8, OBP3-responsive protein1; Zinc finger CCCH domain containing protein 29; carbonic anhydrase 1; late embryogenesis abundant hydroxyl proline-rich glycoprotein; and copper transport family protein*) were up-regulated and six genes (*expansin-like B2 precursor; wound-responsive protein; histone H1.1; short-chain dehydrogenase/reductase* (*SDR*) *family protein; and EFE and GAMMA-VPE*) were down- regulated (Additional file 7: Table S3). *Actin2* was chosen as the reference gene for normalization data. In most cases, the expression values were a little higher than those obtained from the Illumina sequencing (Figure 12).

### Early wounding-responsive genes that act in the first line of the wounding signaling cascade as assessed by qRT-PCR

**Figure 13:**
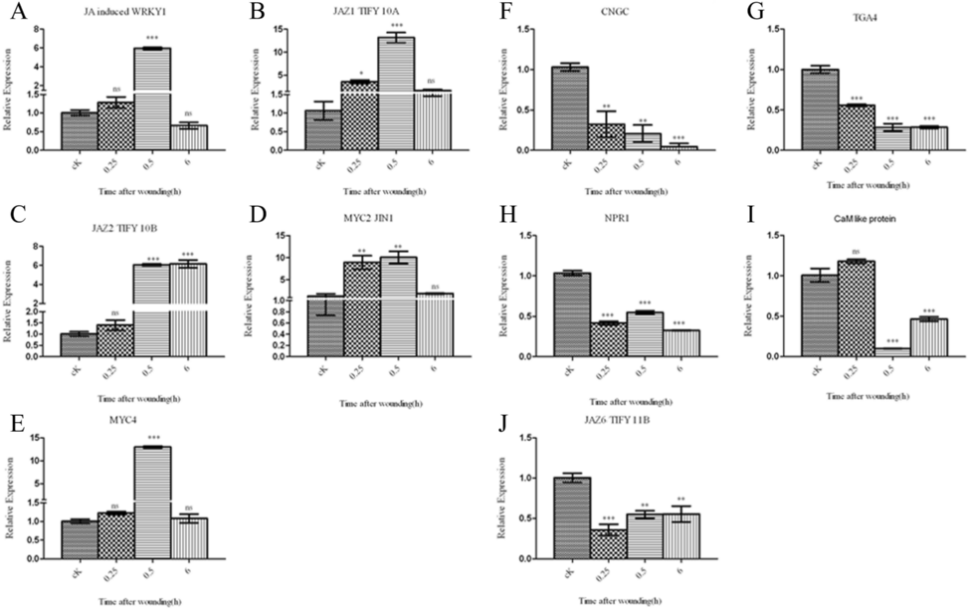
**Temporal expression patterns of the DEGs in sweet potato leaves damaged by MW.** Relative gene expression measured by qRT-PCR at 0.25, 0.5 and 6 h, comparing untreated leaves (cK) and leaves damaged by MW. **(A-E)** Up-regulated genes: JA-induced *WRKY, MYC2, MYC4, JAZ1-TIFY-10A, and JAZ2-TIFY-10B*. **(F-J)** Down-regulated genes: *CNGC, CaM, NPR1, TGA,* and *JAZ6-TIFY-11B*. Error bars indicate the standard deviations for the means of triplicate samples, and the *Act2* gene was used for data normalization. Asterisks indicate statistically significant differences. (Dunnett’s multiple comparison test,* represents P < 0.05; ** represents P < 0.01; *** represents P < 0.001; ‘ns’ not significant represents P > 0.05). The results are representative of three replicates.

Based on our computational analysis, we have selected genes related to the plant-pathogen interaction pathway and plant-hormone signal transduction, and we discovered 10 differentially expressed, early wounding-responsive genes which may act in the first line of the wounding signaling cascade, including *cyclic nucleotide- gated ion channel* (CNGC)*, calmodulin* (*CaM*), *JA-induced WRKY, MYC2, MYC4, JAZ1, JAZ4, JAZ6, non-expressor of pathogenesis related* (*NPR1*)*,* and *TGA*. We compared the time-dependent changes in the expression of these genes with qRT-PCR (Figure 13). The results showed that *JAZ1/TIFY 10b, JAZ2/TIFY 10a, MYC2* and *MYC4* were significantly up-regulated at early time points and these up-regulated genes were associated with wounding or the JA- mediated signaling pathway. Therefore, the induced expression of *JAZ1/TIFY 10b, JAZ2/TIFY 10a, MYC2* and *MYC4* confirms that the first line of the signaling cascade requires JA signaling in the control of the defense response.

**Figure 14:**
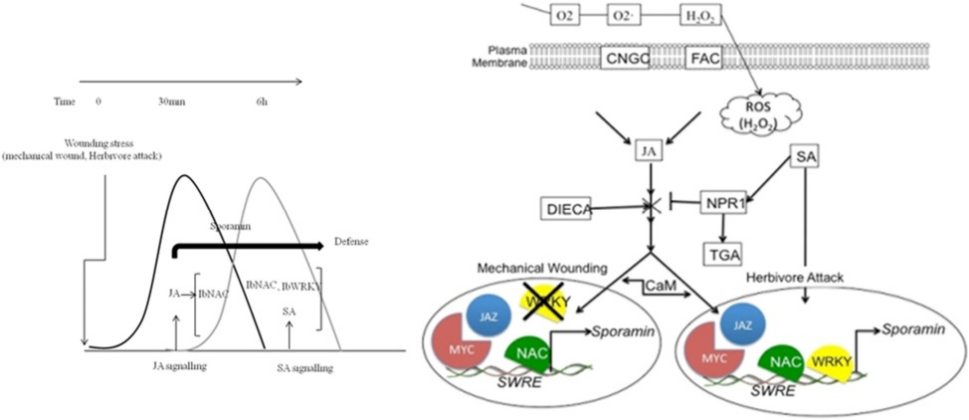
**Proposed model for sporamin gene regulation in sweet potato leaves.** Two mechanisms induced by wounding were clarified in this work: activated by abiotic MW or biotic HA. Our findings illustrate that both of them have an impact on different ROS levels and antioxidant mechanisms, which indicates signal components are different. The results presented two wound–induced signals: MW or HA can change the endogenous level of JA or SA to regulate sporamin. MW was shown to be JA-mediated, signaling to regulate the transcription factor IbNAC1 for the activation of *sporamin* gene expression. On the other hand, HA was shown to be both JA- and SA-mediated, signaling to regulate transcription factors both IbNAC1 and IbWRKY1 for the activation of *sporamin* against herbivores. This antagonistic difference was further confirmed through the analysis of genes identified in the transcriptome and biological network in the context of MW stress. The selected genes involved in the first line of signaling cascade supports further the partial cross-talk between JA- and SA-mediated signal pathways in regulation of *sporamin* gene expression.

On the other hand, the down-regulation of *CNGC* and *CaM* in response to wounding suggests their roles as negative regulators of defense gene expression in the wounding response. It is possible that Ca^2+^/*CaM* control downstream defense genes in the wounding signaling pathway. In addition, we examined *NPR1* and *TGA*, which were down-regulated in response to wounding, which suggests the *NPR1*- and SA-dependent stimulus pathway (Figure 14).

## Discussion

To minimize the damage caused by either MW or HA, plants produce antioxidant and ROS scavenging enzymes [27,28]. Different patterns of ROS and antioxidant mechanisms exist between MW and HA in the sweet potato leaf (Figure 1). This study supports the notion that two different stresses (MW and HA) generate ROS in the plant cells. During this process, production of ^1^O_2_ initiates lipid peroxidation to disrupt PSII as a result of plasma membrane NADPH oxidase (Figure 2) [29,30]. The enhanced activity of SOD in response to MW constitutes the primary defense that efficiently protects membrane lipids against oxidative damage [31]. In parallel, MW reduced CAT activity that can be explained by its interaction with SA, and induced the role in plant-pathogen defense mechanism [28]. However, the increase in CAT activity upon HA may prevent excess H_2_O_2_ production, which is responsible for membrane lipid peroxidation [32]. Increasing evidence of differential activation of these antioxidant enzymes was solely dependent upon growth and development [33].

Sweet potato has differentially and chronologically expressed antioxidant enzymes when under MW and HA stress. The antioxidant system protection against wounding stress depends on growth stages. SOD acts as a primary defense signal for MW at all time points tested. Conversely, CAT and POD followed a two-step activation upon MW and HA. At an early time point, CAT was deactivated upon MW stress. In this instance, accumulation of hydrogen peroxide acts as a signal to induce octadecanoid signaling transduction pathway. In contrast, CAT is activated after prolonged stress caused by HA, and leads to a decomposition of excess H_2_O_2_ at an early time point. At the later time point, both CAT and POD are deactivated. Accumulated H_2_O_2_ acts as a signal to induce octadecanoid and other defense signal transduction pathways (Figures 1 and 2) [33-36]. Furthermore, our study presented evidence that a specific level of JA and SA production induces the expression of defense-related genes that are involved in the antioxidant and detoxification processes (Figure 3) [28,37,38].

### Systemin and JA-mediated signal in sweet potato leaf after MW and HA

We investigated the critical role of phytohormones in orchestrating *sporamin* gene expression in response to MW and HA. The key observation underlying the experiments described is that endogenous JA level was enhanced after MW on the leaf to mediate the signal transduction and trigger the activation of the octadecanoid pathway (JA pathway) during early wounding time points (Figure 3) [39,40]. However, upon HA, the enhanced endogenous SA level could be due to larval oral secretion (violicitin), which interacts with endogenous phytohormones to elicit the SA burst and attenuates JA (Figure 3) [40,41]. In agreement with previous studies of *sporamin* induction after MW or HA (Figure 4) [42,43], our report presented evidence that the *sporamin* is wound-inducible and activated through a systemin-mediated JA-signaling pathway [44]. Although JA is necessary for *sporamin* expression in both MW and HA, while SA was shown to be antagonistic to *sporamin* expression in sweet potato (Figure 5). These differences in cross-talk were consistent with previous reports of MW and HA in leaves, as they are solely dependent on timing, concentration of endogenous signal, and the regulation of defense gene expression. [16,17,39,45-47].

### Wounding signal transduction is mediated by two transcription factors to regulate sporamin gene expression differentially

Many wound-responsive promoters in plant species have been functionally characterized, and several putative wound-responsive *cis*-elements have been identified such as *G-box*[48], *GCC box*[49], *AG-motif*[50,51], *Jasmonate/elicitor responsive element*[52,53], *13-bp/L-box*[54], *W-box*, [53] and *sporamin*[42]. In the present work, we describe the isolation and characterization of two novel transcription factors that bind within the 38-bp -1103- ACATTTCTCGTAAATACGTACAATATCCTTGTCTTTCC -1061- of the *SWRE* region of the *sporamin* promoter of the sweet potato (Figure 4). Furthermore, we identified signal cross-talk between JA and SA signaling pathways that were mediated by Ib*NAC*1 and Ib*WRKY*1 differentially (Figures 5 and 6). It appears that minor variations in the core sequences of *sporamin* promoter impart responsiveness to these different stimuli. Upon MW, IbNAC1, acting as an early transcriptional factor, functions in the JA signal pathway to regulate *sporamin* gene induction in the sweet potato leaf (Figures 5B and 6B) [55]. However, IbNAC1 may partially function in the SA pathway, which harmonizes the action of other downstream defense genes (e.g., *PR* genes) (Figure 6H) [56]. Similarly, induction of Ib*NAC*1expression upon HA explains its role as a convergence transcriptional activator, functioning in both JA and SA signaling pathways to regulate *sporamin* gene expression in the sweet potato leaf (Figure 8B) [55,56]. In our study, we were puzzled to find that Ib*WRKY* is not a positive regulator for *sporamin* gene expression in MW or SA signaling (Figures 5C, 6G and I). However, the MeJA/SA -mediated inhibition of Ib*WRKY* expression at an early time point suggested that endogenous JA level might play an important role in controlling its expression (Figure 6C and I). Moreover, the expression of Ib*WRKY*1 after 6 h upon exogenous MeJA or SA treatment (Figure 6C and I) was a consequence of re-methylation of JA for a positive feedback effect on SA biosynthesis [42,57-60]. This can be tested by quantifying the individual metabolites of JA and SA biosynthesis, and remains to be elucidated.

Interestingly, the strongly up-regulated expression of Ib*WRKY*1 following HA (Figure 8C) suggested, in this instance, at least two steps of regulation were imposed: firstly, an early response of Ib*WRKY,* possibly under the control of *S. littoralis*-specific oral secretion that elicits SA burst. This holds true because the feeding by *S. littoralis* caused more of a marked increase of endogenous SA than the endogenous JA concentration (Figure 3). Secondly, the impact of leaves continuously damaged by S. littorais*,* different from ‘single’ wounding events, accumulates Ib*WRKY*1 expression possibly by FACs, through which JA level was maintained [60,61]. In light of this consideration, Ib*WRKY*1 played a direct role in the defense against herbivore and, indirectly, maintained JA level for *sporamin*- activated defense.

To make a comparison for the cross-talk differences between the two transcription factors IbNAC and IbWRKY in JA and SA signaling, we inferred from the publication and evidence by Li et al. (2004) that the inactivated WRKY70 upon JA signaling to the promoter region of JA- responsive gene was likely controlled by cytosolic NPR1protein. Needless to say, IbWRKY could be analogous to WRKY70 as they reflect a similar cellular SA and JA balance. Therefore, the fate of IbWRKY regulates the *sporamin* gene expression via integrating SA and JA-signaling events, depending upon the stress. In support of this assumption, IbWRKY and WRKY70, an uncommon member of the WRKY transcription factor, are pivotal integrators of the SA and JA signals to regulate plant defense responses.

In contrast, our IbNAC is strictly responsive to both wounding stresses (MW or HA) involved in both JA- and SA- signaling pathways, which suggests it is a positive transcriptional activator for regulating *sporamin* gene expression and partially other downstream genes (e.g. *PR*). Upon prolonged HA feeding, NPR1 is not analogous to IbNAC but acts as an amplification loop for IbWRKY and *PR* gene expression through the SA-mediated independent pathway to defend against additional entry of pathogen signal [57]. In conclusion, our results support the need for future work on investigating the mutants of Ib*NAC*1 and Ib*WRKY*1 and their respective roles.

### Global gene expression profiles in sweet potato response to MW stress

Based on the previous literature, MW stress often overlapped with multiple stress responsive genes and shared between different plant species [13,62-64]. The sweet potato is a non-model organism that has not been extensively studied. Among the contig annotated, we found the majority of DEGs involved in the ‘response to stimulus’ and ‘response to stress’ were related to pathogen, wounding, and abiotic and biotic stresses (Additional file 5: Figure S2 and Additional file 6: Figure S3). Based on the KEGG pathway (Figure 11), we found that the majority of metabolic pathways genes were enriched and are common for sweet potato, suggesting they are involved in cell wall biosynthesis, inter-conversion of nucleotide sugars, biosynthesis of lignin and other cell-wall-bound phenolic compounds at the site of leaf damage caused by MW [65,66]. On this basis, we integrated the results from this study and other published studies and formed a working model for the activation of *sporamin* gene expression and its regulation in response to external stimuli (Figure 14). MW or HA activates JA production, which in turn independently activates the expression of Ib*NAC* and *sporamin* through a JA-mediated signaling pathway. Once the JA signal reaches its threshold level, genes encoding JA biosynthetic enzymes, which are tightly regulated by JA-responsive transcription factors (TFs), are up- or down-regulated. The activation of IbNAC1 binds to the *SWRE* and induces transcription of the *sporamin* gene during early response of wounding stress. The negative cross-talk role of Ib*WRKY*1 specifically functions in SA- signaling and has an indirect role in JA- signaling pathway for the persisting expression of *sporamin*.

In contrast, HA results in an increased biosynthesis of both SA- and JA- signaling and leads to the subsequent activation of parallel signaling pathways. In this context, both IbNAC1 and IbWRKY1 coordinate with each other and bind to the *SWRE* to activate the expression of the *sporamin* gene for defense. Under prolonged HA, IbWRKY1 possibly interacts with FACs, which is necessary for maintaining the early action of *de novo* JA biosynthesis in the leaves. Therefore, Ib*WRKY*1 is involved in both pathways upon HA, and requires the initial signal strength (either extremities of high or low) for the mutual antagonistic interaction of the SA- and JA-mediated signaling to regulate *sporamin* gene expression for defense.

Furthermore, the selected genes involved in the first line of wounding signaling cascade support the partial cross-talk between JA- and SA-mediated signal pathways in the regulation of *sporamin* gene expression. For correlation, the *sporamin* promoter region also has a *G-box-like element* and *GC-core-like element*, indicating that there is room to directly target MYC/JAZ interactions. Similarly, the binding site of *W-box* for IbWRKY1 was controlled by calmodulin through other TFs (e.g., WRKY/TGA) of downstream defense genes. Thus, the wounding-induced expression of sweet potato sporamin differs between MW and HA due to the *cis*-elements of the *sporamin* promoter. However, this is regulated by Ib*NAC*1 and Ib*WRKY*1 and depends on the JA and SA pathways (Figure 14) [67].

## Conclusion

Two mechanisms induced by wounding were clarified in this work: differentially activated by abiotic MW or biotic HA. Our findings illustrate that both of them have an impact on different ROS levels and antioxidant mechanisms, which indicates that signal components are different. The results showed that the two wound–induced signals, MW or HA, can change the endogenous level of JA or SA to regulate *sporamin*. MW was shown to be JA-mediated, signaling to regulate the transcription factor IbNAC for the activation of *sporamin* gene expression. On the other hand, HA was shown to be both JA- and SA-mediated, signaling to regulate transcription factors of both IbNAC1 and IbWRKY1 for the activation of *sporamin* against herbivores. This antagonistic difference was further confirmed through the analysis of genes identified in the transcriptome in the context of MW stress. The selected genes involved in the first line of signaling cascade supports further the partial cross-talk between JA- and SA-mediated signal pathways in regulation of *sporamin* gene expression. The knowledge gained from this study holds great potential for sporamin research within the wounding signaling pathway in the near future.

## Methods

### Plant material and growth conditions

Sweet potato (*Ipomoea batatas* cv. Tainong 57) plants were grown in pots at 28°C under a 16-h light (2000 lux)/8-h dark (16 L/8D) cycle. Sweet potato with at least five fully expanded leaves per pot (n = 3) were utilized for each experiment, and each data set was from three biologically independent replicates.

### Antioxidant enzyme extraction and activity assay

The third sweet potato leaf was collected at various time points (15 min, 30 min and 6 h) after MW and HA and frozen immediately in liquid N_2_ (as described above). For enzyme assay, 0.3 g of leaf was ground with 3 mL of ice-cold 50 mM sodium phosphate buffer (pH 7.5). Enzyme assays were then performed immediately. Total phenolic and DPPH radical scavenging assays were performed spectophotometrically [68,69]. The extraction buffer for estimating total phenolic content contained 0.5 mL of sodium carbonate (5% w/v) and the solution of gallic acid (0–0.1 mg/mL) in ethanol (95%). For DPPH, the extraction buffer contained the solution of ascorbic acid (5–25 μg), 0.1% DPPH solution [69]. The extraction buffer for SOD (EC 1.15.1.1) contained 100 μL of 50 mM potassium phosphate (pH 7.8), 0.1 mM Na_2_EDTA, 14 mM- methionine, 75 μM -NBT and 2 μM – riboflavin [70]. The extraction buffer for CAT (EC 1.11.1.6) contained 0.6 mL potassium phosphate buffer (pH7.4), 0.5 mL hydrogen peroxide (H_2_0_2_) (molar extension coefficient: 44 M^-1^ cm^-1^) [71]. For POD (EC1.11.1.7) and H202 assay, the extraction buffer contained 0.05 mL of guaicol solution (20 mM) and 0.03 mL of 12.3 mM H_2_0_2_. Hydrogen peroxidase activity assay was performed using the TMB method. The enzyme extract buffer contained 20 mM TMB dissolved in 95% ethanol, 600 uL of 0.1 M acetate/citrate buffer (pH 4.5), 100 μL of 100 μM of H202, 100 μL of 0.5 M H_2_SO_4_ and 100 μL [34,72].

### Fluorescence detection of singlet oxygen species generation in response to wounding

Fully expanded, intact third leaves were wounded by MW or HA at 0.25 h. SOSG (Invitrogen) was dissolved in methanol at a final concentration of 50 μm in 50 mM sodium phosphate buffer (pH 7.4), according to the manufacturer’s instructions. Unwounded (cK) and wounded leaf was slowly infiltrated with 50 μm SOSG either in the dark or light (150 μmol m^-2^ s^-1^ PPFD) for 30 min. The leaves were dried in the dark and then photographed using CLSM with excitation at 475 nm and emission at 525 nm [73].

### Phytohormone analysis of JA and SA by LC-ESI-MS

Unwounded (cK), MW and HA sweet potato 3rd leaf were sampled at 0.25, 0.5 and 6 h and frozen immediately at -80°C. The samples (100 mg) were immediately ground to a fine powder in liquid N_2_ and then exposed to extraction buffer (100 μl of 100% methanol). The samples were centrifuged with 1 mm and 2 mm (1:1) zirconium oxide beads by vortexing at 16,000 × g for 10 min. After centrifugation at 16,000 × g for 10 min, the supernatant was collected for LC-ESI-MS analyses based on the procedure [74].

### Construction and screening the one-hybrid library and screening interacting proteins

A total 950 μg of RNA from sweet potato 3rd leaf 0.5 h and 1 h after wounding was used to extract poly (A)^+^ RNA by Oligotex mRAN Midi Kit (QIAGEN). Briefly, aliquots of poly (A)^+^ RNA were reverse-transcribed separately by using either random or oligo (dT) primers, and the cDNA products were combined and inserted in the shuttle vector, *pGADT*7*-Rec*2, containing GAL4activation domain (p*GAL*4*AD*). Yeast one-hybrid reporter (Matchmaker™ One-Hybrid Library Construction & Screening Kit) construct was prepared by cloning tandems containing three repeats of cis-elements of *sporamin* promoter (*3*x*SWRE* (38-bp)-1103- ACATTTCTCGTAAATACGTACAATATCCTTGTCTTTCC -1061) was used as a bait sequence, and inserted in reporter plasmids p*HIS*2 to select the DNA-binding domain encoded in the *sporamin* cDNA library. The p*HIS*2 reporter vector of *3*x*SWRE*, cDNA and p*GADT*7-*Rec*2 vectors were co-transformed to the yeast strain Y187 lacking His-, Trp-, and Leu-. The transformed yeast cells were spread on SD/His/-Leu/-Trp in the presence of 40 mM 3-AT. Transformation efficiency was carried out by LiCl- polyethylene glycol, and transformants grown on SD/-Leu, SD/-Trp were tested for *β-galactosidase* activity. Plasmids from putative positive clones of 300–400 colonies appeared on SD/-His/-Leu/-Trp +40 mM 3-AT plates were isolated after three days of incubation. The 252 colonies were analyzed by colony PCR to amplify the cDNA insert. Ninety-four amplified cDNA inserts were sequenced, among them two cDNA with putative domains as conserved, namely NAC and WRKY. To eliminate false positive colonies, these plasmids were separately introduced to the yeast cells containing either *3*x*SWRE* or p*GADT*7-*Rec*2- IbNAC/IbWRKY grown in the SD/-His/-Leu/-Trp + 100 mM 3-AT selection medium.

### MW, application of hormone and hormone inhibitor and herbivore feeding experiment

The wounding of the leaf was carried out as follows. The third leaf counted from the top of the apical meristem was wounded by squeezing with forceps (one time) and then collected after 0.25, 0.5 and 6 h. Plants with fully expanded six to eight leaves per pot were sprayed with the following chemicals: MeJA, SA and diethyldithiocarbamic acid (DIECA). The chemical and concentrations were used as follows: MeJA (0.05 mM) dissolved in 75% ethanol, SA (2 mM) dissolved in water and DIECA (1 mM) dissolved in water. A third leaf was collected at 0.25, 0.5 and 6 h after treatment. For control, tissues were sprayed with water. All the chemicals were purchased from Sigma. For insect feeding, second instar larvae of *Spodoptera littoralis* were starved for 2 days and subsequently allowed to feed on the sweet potato leaves. Approximately 7 to 10 second instar larvae per pot were placed on the upper side of the third intact leaf and were observed until they began feeding [75]. The larvae that failed to feed on the leaves within 30 min were discarded. The leaves eaten by the larvae were collected at different time points, 0.25, 0.5 and 6 h, and stored at -80°C until use. Larvae were weighed on an analytical balance. Feeding was allowed for 7 days, and the masses of the leaves consumed by the larvae and masses of the larvae were recorded for every other day for 7 days. Each trial had a total of 10 second instar larvae (n = 30) and was repeated three times. Mortality and longevity of the larvae were recorded daily. On day 7, all the live larvae were weighed and mortality was recorded.

### QRT-PCR

Total RNA was prepared from sweet potato leaf tissues using the pine tree method. [76] The quality of the RNA was assessed using Tecan Nanodrop analyzer (Tecan group Ltd, USA, San Jose). The A260/A280 ratios of the RNA samples, which were dissolved in 10 mM Tris (pH 7.6), ranged from 1.9 to 2.1. Total RNA (5 μg) was used to synthesize cDNA following the instructions of the First Strand cDNA Synthesis Kits (Fermentas). Completely synthesized cDNA was utilized for RT-PCR using specific primers. The primer sets used are listed as follows: *SPOR* F’-ATTAGTTTGGACGGGCG’, R-‘TTAACGCCGCCGTTAAGG’; Ib*NAC*1 (Accession:GQ280387) (F‘CGGCCGGGGATACAAATTTGTAAGCTT‘, R-‘GAATCGGAATCCCGGCGGCATCTC‘); Ib*WRKY*1 (Accession: GQ280386) (F-‘GGAATTGAGATGGCTGTAGAGTTGTTG‘,R-‘GAAAACCCACTAACTTGCGGCTGAGA‘) and *Ubiquitin* (*UBQ*) F-(‘CAACAACACTGCCGTCGC’, R-TCTTCTCCTTCATCATCGCCAC‘).

### Illumina cDNA library preparation and sequencing

Total RNA was extracted from each leaf sample using a previously described Cetyl trimethylammonium (CTAB) procedure (see above). The *A*260/*A*280 ratios of the RNA samples, which were dissolved in 10 mM Tris (pH 7.6), ranged from 1.9 to 2.1. For cDNA synthesis and Solexa sequencing, 20 μg total RNA was used at a concentration of ≥400 ng/μl. Poly(A) mRNA was first purified using oligo(dT) beads then the mRNA was fragmented into small pieces (100–400 bp) using divalent cations at an elevated temperature. The cleaved RNA fragments were then primed with random hexamers and used to synthesize first-strand and second-strand cDNA. The cDNA fragments that were >200 bp in size were selected by agarose gel electrophoresis and enriched by PCR amplification. Finally, two single-end cDNA libraries were constructed for sequencing on an Illumina (GA) IIx platform.

### Pre-processing and quality assurance of sequencing reads

The reads delivered by the Beijing Genomics Institute (BGI) were processed using the following procedure to acquire qualified reads. First, the reads containing adapter sequences or including more than 10% uncertain nucleotides (Ns) were excluded. Next, a read was further excluded if 50% of its bases were low quality (quality score <5, Illumina GA Pipeline v1.3).

### *De novo* assembly

The qualified reads of wound-stress and control samples were respectively assembled into distinct contigs using SOAP *denovo* software ( http://soap.genomics.org.cn), which applies the de Bruijn graph data structure to construct contigs (assembled sequences). All of the parameter settings followed the standard procedures suggested by BGI. Next, the two sets of contigs were integrated into one ‘unigene set’. The sequences of this ‘unigene set’ were used as the reference sequences in the subsequent analysis of gene quantification [77].

### Gene annotation of the assembled sequences

The assembled sequences were annotated using a previously described procedure involving sequential BLAST searches (Altschul, Madden, Schaffer, Zhang, Zhang, Miller & Lipman, 1997), which are designed to find the most descriptive annotation for each sequence suggested by standard BGI analysis. The assembled sequences were compared with protein sequences collected from GenBank’s non-redundant database [ http://www.ncbi.nlm.nih.gov/RefSeq/] using the BLASTx algorithm. In the BLASTx analysis, the e-value cutoff was set as 1e^-10^.

### Quantification and differential expression analysis of genes

All filtered reads were mapped to the reference sequences and then the accumulated read counts were normalized as the number of mapped reads in a given contig per kilobase of contig length per million mapped reads (RPKM) [78]. To identify the genes stimulated by wounding stress, the expression values of each gene in the two samples were estimated. The statistical significance of the differential expression of each gene was determined using the method described [79], and the results of the statistical tests were corrected for multiple testing with the Benjamini-Hochberg FDR. Sequences were deemed to be significantly differentially expressed if the adjusted *P* value was <0.001 and there was at least a two-fold change (>1 or < -1 in the log2 ratio value) between the two samples. All of the procedures followed the standard BGI analysis.

### Pathway and GO enrichment analysis

The KEGG, the major public pathway-related database, was employed to identify the metabolic or signal transduction pathways containing the DEGs from the whole genome background using a hypergeometric test [80]. The same calculation was applied for GO enrichment analysis and both analyses were incorporated into the standard BGI analysis.

### Validation of DEGs by semi-quantitative and quantitative real time-PCR (qRT-PCR) analysis

Total RNA was isolated from control and wounded (15-min) samples of fully expanded third leaf tissue. First, we performed semi-quantitative RT-PCR analysis for 15 genes and submitted them for re-sequencing analysis to confirm the reliability of the Illumina sequencing. Subsequently, 15 genes were subjected to validation by qRT-PCR. The temporal expression pattern of the remaining 10 genes was subject to study at various time points as described above. All primer pairs are listed in the Additional file 8: Table S4. Quantitative RT-PCR was performed with an ABI 7500 Real-Time PCR system in a final reaction volume of 20 μl containing 5 μl diluted cDNA in water, 10 μl 2× SYBR Green Real-Time PCR Master Mix (A) and 10 μM each of forward and reverse primers. The thermal cycling conditions were as follows: 40 cycles of 95°C for 3 sec for denaturation and 60°C for 45 sec for annealing. QRT-PCR was performed for three biological replicates. *Actin2* (*Act2)* was used to normalize gene expression. The relative expression levels of the genes were calculated using the 2^-△△CT^ method [81], which determines the difference in the CT between the control *Act2* product and the target gene product. Relative fold change for each gene was compared to control, which was set to one.

### Statistical analysis

All statistical analyses were performed using GraphPad Prism 5.0 (GraphPad Software, La Jolla, CA, USA). Biochemical analysis, the quantification of phytohormones, was performed by two-way analysis of variance (ANOVA) (Bonferroni post-test). One-way ANOVA followed by Dunnett’s Multiple Comparison Test at 95% confidence intervals) was performed for gene expression. Bar represents mean (SE±).

## Competing interest

The authors declare that they have no competing interest.

## Authors’ contributions

YKW, CYC coordinated and participated in designing the experiments. RSK conceived, implemented and performed the experiments. IWL assisted one- hybrid assay and screening was performed by RSK and IWL. RSK, CYC and MJC participated in the computational analysis. MJC performed the bioinformatics analysis and helped in interpreted of the results. RSK wrote the manuscript. CYC and YKW critically revised the complete structure and organization of the manuscript. All the authors have read and approved the final version of the manuscript.

## Supplementary Material

Additional file 1: Figure S1 Distribution of total clean reads and its coverage A Control Library B Wouding Library. Number of clean reads and its gene coverage in the range of percentage. Click here for file

Additional file 2: Table S1 Statistical analysis of transcriptome sequencing and *denovo* assembly. Click here for file

Additional file 3: Table S2 Gene enrichment analysis of sweet potato DEGs compared with *Arabidopsis*. Click here for file

Additional file 4 Differential gene expression profiling of sweet potato in response to mechanical wounding (MW). Click here for file

Additional file 5: Figure S2 Heatmap illustrates the differentially expressed genes that were related to ‘response-to-stimulus’ and ‘respose-stress’ in response to MW. Click here for file

Additional file 6: Figure S3 Heatmap illustrates the differentially expressed genes that were related to biotic and abiotic stimulus in response to MW. Click here for file

Additional file 7: Table S3 List of 15 DEGS genes selected for qRT-PCR and fold change. Click here for file

Additional file 8: Table S4 List of primers used for qRT-PCR. Click here for file
