# Supplementary figures and images for: Differential activation of sporamin expression in response to abiotic mechanical wounding and biotic herbivore attack in the sweet potato
Source: BMC Plant Biol. 2014 Apr 28;14:112. doi: 10.1186/1471-2229-14-112 (PMC4108030; doi:10.1186/1471-2229-14-112)

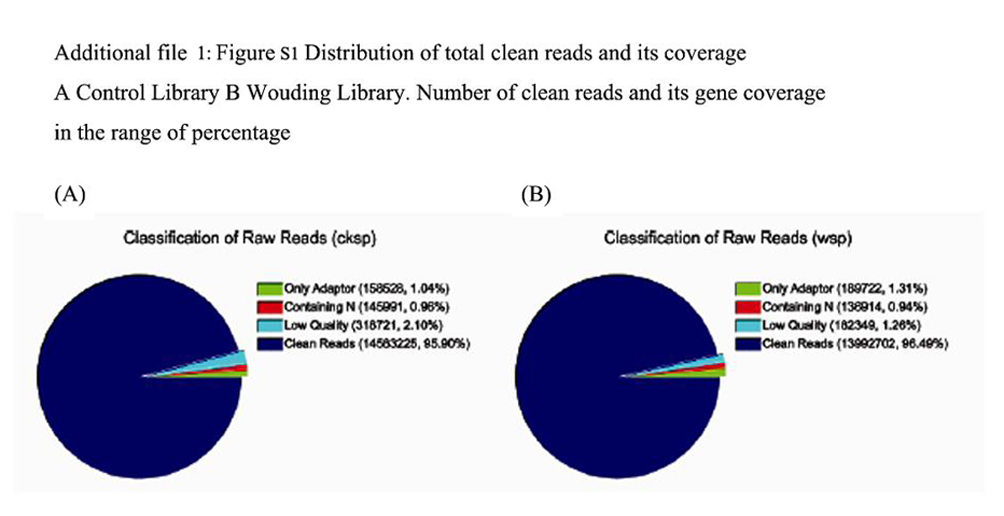

Supplement: Additional file 1: Figure S1 — Distribution of total clean reads and its coverage A Control Library B Wouding Library. Number of clean reads and its gene coverage in the range of percentage. [file 1471-2229-14-112-S1.tif]

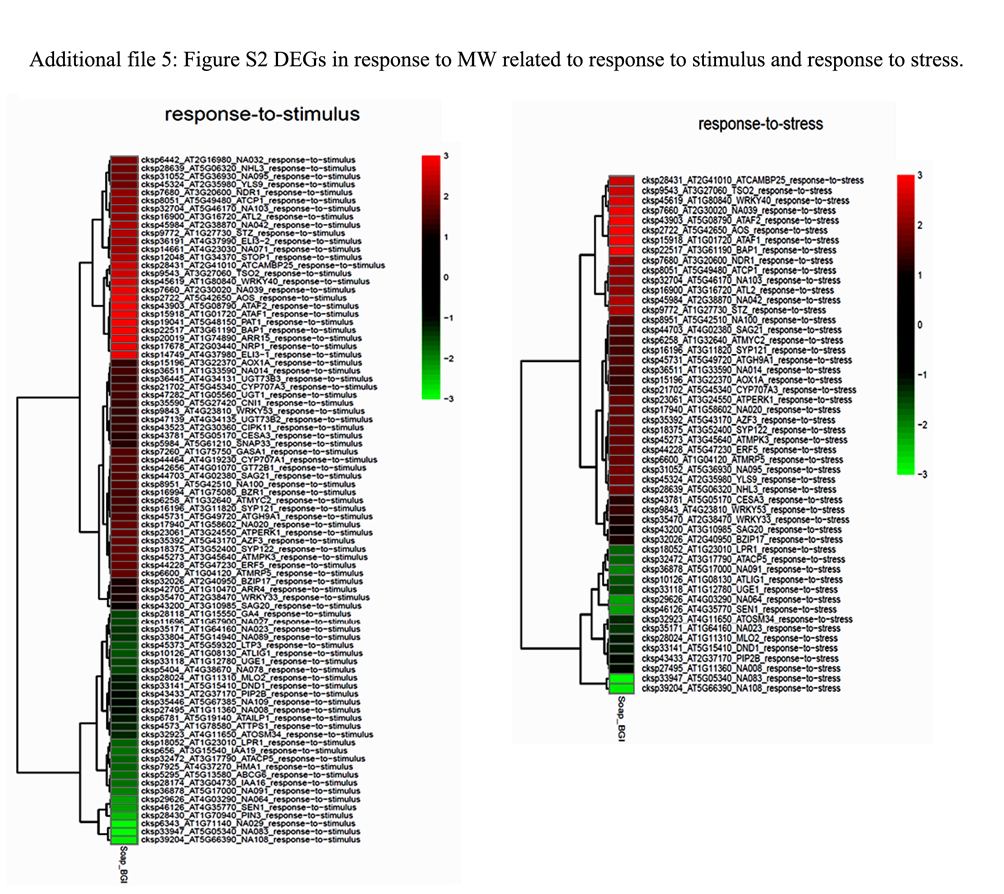

Supplement: Additional file 5: Figure S2 — Heatmap illustrates the differentially expressed genes that were related to ‘response-to-stimulus’ and ‘respose-stress’ in response to MW. [file 1471-2229-14-112-S5.tif]

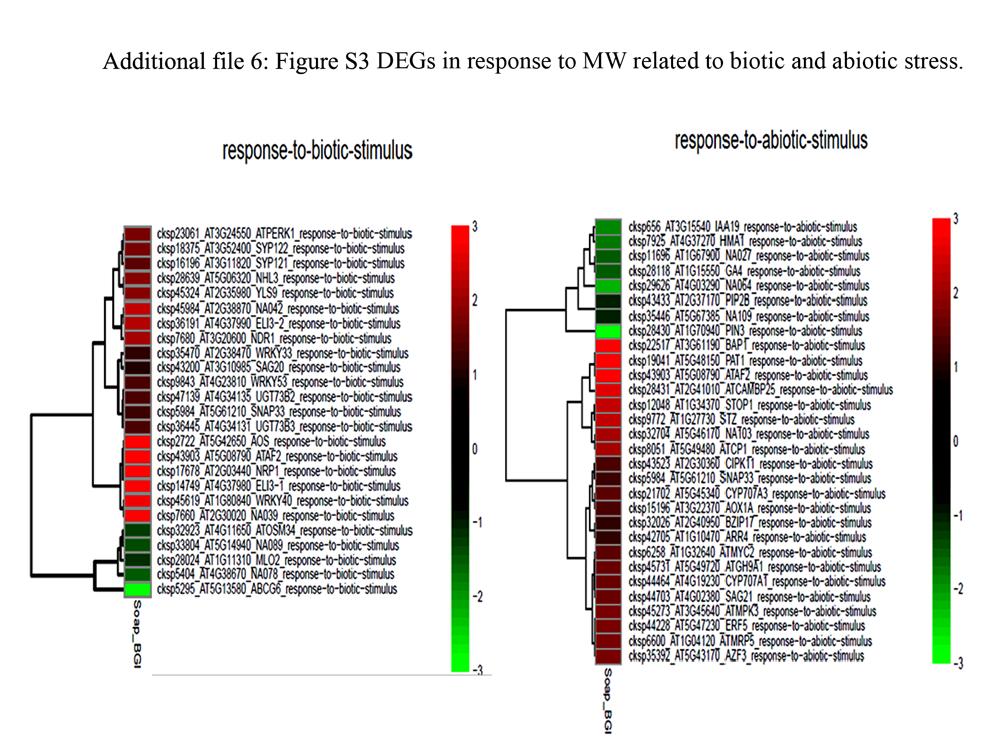

Supplement: Additional file 6: Figure S3 — Heatmap illustrates the differentially expressed genes that were related to biotic and abiotic stimulus in response to MW. [file 1471-2229-14-112-S6.tif]
