# Supplementary material for: Differential activation of sporamin expression in response to abiotic mechanical wounding and biotic herbivore attack in the sweet potato
Source: BMC Plant Biol. 2014 Apr 28;14:112. doi: 10.1186/1471-2229-14-112 (PMC4108030; doi:10.1186/1471-2229-14-112)
Supplement: Additional file 2: Table S1 — Statistical analysis of transcriptome sequencing and denovo assembly. [file 1471-2229-14-112-S2.doc]

**Additional file 2: Table S1**

| **Statistical analysis of transcriptome sequencing and *denovo* assembly** | | | |
| --- | --- | --- | --- |
|  | **Cksp** | **Wsp** | **Unigene set** |
| Contig count | 48,839 | 45,722 | 41,806 |
| Total read count | 20,777,585 | 16,098,635 | - |
| Mean read length | 75 | 76.15 | - |
| Total read length | 1,558,374,757 | 1,225,914,651 | - |
| Mean contig length | 519 | 493 | 561 |
| Total contig length | 25,368,159 | 22,571,391 | 23,443,573 |
| GC content in % | 45 | 45 | 45 |
