# Supplementary material for: Differential activation of sporamin expression in response to abiotic mechanical wounding and biotic herbivore attack in the sweet potato
Source: BMC Plant Biol. 2014 Apr 28;14:112. doi: 10.1186/1471-2229-14-112 (PMC4108030; doi:10.1186/1471-2229-14-112)
Supplement: Additional file 3: Table S2 — Gene enrichment analysis of sweet potato DEGs compared with Arabidopsis. [file 1471-2229-14-112-S3.doc]

**Additional file 3:** Table S2 Gene enrichment analysis of sweet potato DEGs compared with *Arabidopsis*

| **GO related** | **Sweet potato** |
| --- | --- |
| # of differential expressed sequences | 1,070  (up: 684; down: 386) |
| # of gene-hit sequences | 809  (up: 497; down: 312) |
| # of Arabidopsis gene | 590 |
| # of genes with BP GO term hit | 415 |
| # of genes with MF GO term hit | 436 |
| # of enriched BP GO terms (p-value < 0.01) | 71 |
| # of enriched MF GO terms (p-value < 0.01) | 44 |
