# Supplementary material for: Differential activation of sporamin expression in response to abiotic mechanical wounding and biotic herbivore attack in the sweet potato
Source: BMC Plant Biol. 2014 Apr 28;14:112. doi: 10.1186/1471-2229-14-112 (PMC4108030; doi:10.1186/1471-2229-14-112)
Supplement: Additional file 7: Table S3 — List of 15 DEGS genes selected for qRT-PCR and fold change. [file 1471-2229-14-112-S7.doc]

**Additional file 7: Table S3. List of 15 DEGS genes selected for qRT-PCR and fold change**

| **Contig number** | **Arabidopsis id** | **Gene description** | **Solexa fold** | **qRT-PCR fold** |
| --- | --- | --- | --- | --- |
| Up-regulated genes | | | | |
| cksp45476 | AT3G46970 | *alpha-glucan phosphorylase 2* | 1.869442 | 0.17364 |
| cksp42579 | AT4G30440 | *UDP-D-glucuronate 4 epimerase 1* | 2.41583 | 4.11245 |
| cksp33249 | AT1G5060 | *Ethylene-responsive transcription factor 3* | 2.471886 | 2.891 |
| cksp6941 | AT2G23810 | *Tetraspanin8* | 2.26549 | 4.404 |
| cksp7191 | AT5G53450 | *OBP3-responsive protein1* | 2.076541 | 1.116 |
| cksp35365 | AT2G40140 | *Zinc finger CCCH domain containing protein 29* | 1.257632 | 4.228 |
| cksp45324 | AT2G35980 | *Late embryogenesis abundant hydroxyl proline-rich glycoprotein* | 1.84389 | 1.82 |
| cksp35374 | AT5G52740 | *Copper transport family protein* | 1.192658 | 3.914 |
| cksp35655 | AT2G40140 | *Carbonic anhydrase 3* | 1.513552 | 2.70692 |
| Down regulated genes | | | | |
| cksp45581 | AT4G30380 | *EXPANSIN-LIKE B2 PRECURSOR* | -1.12541 | -0.224 |
| cksp43248 | AT4G28240 | *wound-responsive protein-related* | -1.83602 | -0.78 |
| cksp7279 | AT1G06760 | *Histone H1.1* | -1.07057 | -1.06 |
| cksp32819 | AT4G13250 | *Short-chain dehydrogenase/reductase SDR* | -2.92154 | -1.05326 |
| cksp43209 | AT1G05010 | *1-aminocyclopropane-1-carboxylate oxidase* | -1.89179 | -1.09349 |
| cksp6627 | AT4G32940 | *cysteine-type endopeptidase* | -1.56017 | -2.20307 |
