# Supplementary material for: Differential activation of sporamin expression in response to abiotic mechanical wounding and biotic herbivore attack in the sweet potato
Source: BMC Plant Biol. 2014 Apr 28;14:112. doi: 10.1186/1471-2229-14-112 (PMC4108030; doi:10.1186/1471-2229-14-112)
Supplement: Additional file 8: Table S4 — List of primers used for qRT-PCR. [file 1471-2229-14-112-S8.doc]

**Additional file 8:** Table S4 List of primers used for qRT-PCR.

| **Sequence id** | **Arabidopsis id** | **Gene symbol** | **Fwd (5’-3’)``** | **Rev (5’-3’)** |
| --- | --- | --- | --- | --- |
| **Up- regulation** | | | | |
| cksp6941 | AT2G23810 | TET8 | CAACGAACTGGACCAAGAC | GGAACACGAGGAAGATGATG |
| cksp35655 | AT3G01500 | CA1 | CCATATTCTTCCTTCTCACTG | CCGTCGTCTTCTTCTTCC |
| cksp42579 | AT4G30440 | GAE1 | GTCGTAGTAGTCGTTGAAG | GGATGTCGGTATTGGTAAC |
| cksp45324 | AT2G35980 | YLS9 | GCTCAGGTTGGTGGTGTTC | CGCATCGGCATCTACTACG |
| cksp35374 | AT5G52740 |  | GAGAAGAAGAAGGACGATGG | ATAATAAGGATGTTGTTGTTGATG |
| cksp35365 | AT2G40140 | CZF1 | TTCCTTCCGTCCCACTCC | CCACCTGCTCCTGATCTATG |
| cksp45476 | AT3G46970 | ATPHS2 | CAGGAGAGCGGCGAAGAG | ATGGAATCAAGAAAGCAAGAAGC |
| cksp7191 | AT5G53450 | ORG1 | ACTGGTTACTGGATAGCC | CAGAGTTGTGATTGGAAGG |
| cksp33249 | AT1G50640 | ERF3 | TCTTCGTCGTATTGGTAAC | ATCTTCGGTCATAGGCATAG |
| **Down-regulation** | | | | |
| cksp7279 | AT1G06760 | histone H1 | GCCGCCGCTGCTAAGAAG | CAATCGCCACTTGACTTGAACC |
| cksp45581 | AT4G30380 | EXLB2 | CAGTGTGCCTTGCTCTTGG | CGTGTGATTCTTGTGAGTTTGG |
| cksp43248 | AT4G28240 |  | TCTTCTTCTTCATCTTCCGCATC | CTCTCCGCTCCCGTTTCC |
| cksp32819 | AT4G13250 |  | CTGGGCTGCGAGAGGTAAC | GGCTTGGAGGAATCATCATTGG |
| cksp43209 | AT1G05010 | EFE | TGATTACAGGAAGGTTATGAAGG | TTGATGAGGTCTGGCTTGG |
| cksp6627 | AT4G32940 | GAMMA-VPE | CATCGGCTTCTCTGTTCTC | TCGTCGCTTCTCTTCTCC |
|  | | | **Fwd (5’-3’)``** | **Rev (5’-3’)** |
| cksp37567 |  | JA-induced WRKY | TTCGGAGGATCGGAGTATTCTTG | CCCAATGAAATTGCTCTCCACTTC |
| cksp33141 |  | CNGC | GTGCACGCAGGTTGGAGTTC | CGAGCGGTGGAAATCTTGTGA |
| cksp6258 | AT1G32640 | MYC2 | TCCCACCATAGCCGCCTTTA | ATGGCGATGGAATTGAAGCAC |
| cksp37632 | AT4G17880 | MYC4 | GTGAGAGAAGCCGACAGCAG | GCCGAGGAGGGACGCTTTGT |
| cksp1944 | AT1G74950 | JAZ2/TIFFY 10B | CTGCGACAATGGATTTTCTGG | GGGAGTGCGGTTGATGTTTAT |
| cksp18573 | AT1G19180 | JAZ1/TIFFY 10A | CTC GCAGACTTGCAGCCTCCT | GCTCAGGGAGGGGATCTGTGG |
| cksp7087 | AT1G72450 | JAZ6/TIFFY 10B | ATGGCGATGGAATTGAAGCAC | TGGCTCAACAGATTGCAGGT |
| cksp45574 |  | TGA | ACAAATCGAGCCCTGACTGA | CCT GCTCCCGCGCTTATC |
| cksp32182 |  | NPRI | CCTTGCTTTGCGATGTACAACA | CAAGGCCAACATCTCATCATCTT |
| cksp4838 |  | CaM | CCCTCTCTCTCTTCTCTCCGACAA | TCCGTCACTACTTGCCCAAT |
| **Internal control** | | | | |
| ACT2 |  |  | ACCGGAATGGTTAAGGCTGGTT | TCCTCTCTTCGACTGAGCTTCATC |
